# Obesity and the Role of Short Duration Submaximal Work on Cardiovascular and Cerebral Hemodynamics

**DOI:** 10.1371/journal.pone.0153826

**Published:** 2016-04-18

**Authors:** Lora A. Cavuoto, Rammohan V. Maikala

**Affiliations:** 1 Industrial and Systems Engineering, University at Buffalo, Buffalo, New York, 14260, United States of America; 2 Providence Regional Medical Center, Providence Strategic and Management Services, Everett, Washington, 98201, United States of America; Kurume University School of Medicine, JAPAN

## Abstract

The objective of this study was to compare gas exchange, cardiac and cerebral hemodynamic responses between 10 non-obese and 10 obese men during submaximal work. With the increasing prevalence of obesity, there is a need to understand the impact of obesity on work-induced responses. Participants completed a step-wise incremental cycling until they reached 60% of their age-predicted maximum heart rate. Gas exchange, cardiac and pre-frontal cortex hemodynamic responses were simultaneously measured during rest, work, and recovery. The non-obese group reached ~43% of their predicted maximal aerobic capacity as compared to ~34% in the obese group, with the non-obese working at a relatively higher workload and for more duration than the obese. The obese had elevated baseline heart rate and reduced whole-body oxygen uptake per body weight at baseline and task termination. Other cardiac and cerebral responses, although increased from baseline, were similar between groups during submaximal effort. In the obese, during recovery oxygen uptake and heart-rate recovery were slowest; cardiac output and rate pressure product were greatest, and left ventricle ejection time was shortest. However, both groups exhibited similar cerebral hemodynamics during recovery. These finding imply that, irrespective of their low aerobic fitness, obesity does not impair myocardial performance and cerebrovascular function during graded submaximal work, however, recovery from a short duration of work was influenced by their fitness level. Since a majority of activities of daily living are performed at individual’s submaximal level, understanding influence of obesity on submaximal work is critical.

## Introduction

With an increasing trend toward sedentary living and the associated prevalence of obesity (defined by standard body mass index (BMI) > 30 kg/m^2^) [1], examining the role of these modifiable personal factors on cardiovascular fitness and responses to submaximal work is critical. However, there has been mixed evidence on the role of obesity on cardiorespiratory fitness and exercise capacity. Typical exercise modes and occupational tasks are most often performed at submaximal levels, therefore it is essential to examine BMI-related differences during submaximal exercise testing. Lafortuna et al. [2] observed a 23% increase in energy consumption for obese compared to non-obese women during submaximal cycling. While levels of deconditioning are still unclear in general, obesity-related compromises in ventilatory mechanics may require adaptations to training modalities for effective and safe exercise or work [3]. Based on treadmill exercise testing, Goran et al. [4] demonstrated a reduced submaximal aerobic capacity in both overweight and obese participants as evidenced by a higher submaximal heart rate, respiratory exchange ratio, and percent maximal oxygen uptake, and shorter time to exhaustion, suggesting that the limiting factor in aerobic-type activities for the obese is not the cardiorespiratory system per se, but rather a limitation in their submaximal aerobic capacity. Nevertheless, the role of obesity as a function of graded submaximal work and exercise tolerance has not been extensively explored.

In a review on control mechanisms of cardiovascular regulation to exercise, Nobrega et al. [5] emphasized the importance of central command, the arterial baroreflex and chemoreflex, and the exercise pressure reflex. At the cerebral level, oxygenation, measured by near-infrared spectroscopy (NIRS), is affected by the level of exercise intensity [6]. For example, Cavuoto and Maikala [7] demonstrated that acute exposure to repetitive lifting exercise decreases cardiorespiratory responses and cerebral hemodynamics in individuals who are obese, contributing to their reduced lifting capacity. An inverse correlation between BMI and prefrontal cortex metabolic activity during cognitive function was demonstrated using brain imaging, suggesting reduced cerebral blood flow in overweight individuals [8, 9]. At the skeletal muscle tissue level, Karabulut and Garcia [10] used limb blood flow restriction technique to demonstrate impaired cardiorespiratory responses in young obese men and women during submaximal constant workload cycling. However, it is not clear if a simultaneous contribution of metabolic regulation at the whole body and tissue level will demonstrate a similar decreasing trend in the obese group as a function of graded submaximal work.

Cardiovascular fitness is also dependent on the ability of the body to recover from exercise. Borresen and Lambert [11] emphasized the importance of training status on autonomic control of heart rate, and reported rapid decrease in heart rate after high-intensity exercise in athletes compared to untrained participants. Most recently, Franco et al. [12] demonstrated that black obese adolescent females have greater sympathetic activity, as evaluated by an exercise recovery index following volitional exhaustion during a graded treadmill exercise (ratio of heart rate and whole body oxygen consumption plateau) than white obese adolescent females. Obesity-related slowing in recovery of oxygen uptake and heart rate may serve as secondary measures of cardiovascular fitness and disease risk [13]. Although a majority of these studies have examined cardiovascular responses following exercise, the influence of submaximal work on subsequent recovery at both whole body and tissue level simultaneously has not yet been extensively explored.

The present study, therefore compared cardiovascular and cerebral hemodynamic responses to submaximal work in a group of young males who were either obese or non-obese. A secondary objective was to investigate the role of obesity in recovery from submaximal work. It was hypothesized that obesity compromises physiological responses during and following submaximal work.

## Methods

### Participants

Twenty healthy young men were recruited into two groups based on their BMI, 10 non-obese (18.5<BMI<25 kg/m^2^) and 10 obese (BMI>30 kg/m^2^). Skinfold thickness and waist to hip ratio were used as secondary measures for confirmation of obesity classification (Table 1). Estimated body fat percentage was determined from the equations presented by Jackson and Pollock [14]. Participants were excluded if they had diabetes, uncontrolled high blood pressure, or any cardiovascular or musculoskeletal disorders that would impair their ability to complete the experiment. All participants were non-smokers who were recreationally active. Study protocols were approved by the New England Institutional Review Board and all participants provided their written informed consent prior to participation.

**Table 1:** Summary of participants’ demographic and anthropometric characteristics in mean (standard deviation).

	Non-obese	Obese	*p* value*
Age (years)	27.2 (5.1)	29.7 (4.4)	0.26
Height (m)	1.76 (0.07)	1.75 (0.05)	0.86
Mass (kg)	69.9 (7.0)	105.3 (11.5)	< 0.001
Body Mass Index (BMI; kg/m^2^)	22.6 (1.5)	34.2 (2.5)	
Body Adiposity Index (BAI; % adiposity)**	21.9 (2.5)	30.9 (1.9)	< 0.001
Body Surface Area (BSA; m^2^)***	1.85 (0.1)	2.26 (0.2)	< 0.001
Waist Circumference (cm)	81.9 (6.6)	110.8 (11.0)	< 0.001
Hip Circumference (cm)	93.5 (4.4)	113.4 (5.6)	< 0.001
Skinfold Thickness (mm)			
Chest	7.9 (5.4)	18.8 (4.4)	< 0.001
Abdominal	12.3 (7.6)	31.3 (5.3)	< 0.001
Thigh	10.1 (6.2)	27.7 (20.8)	0.027
Predicted body fat (%) ****	9.3 (5.1)	21.9 (5.6)	< 0.001

* *p* value is the result of the t-test comparing the groups

** calculated as $\frac{100\times hip\;circumference}{height\times \sqrt{height}}-18$

*** calculated as $\sqrt{weight\times \frac{height(cm)}{3600}}$

**** calculated based on Jackson and Pollock [14]

### Procedures

**Fig 1:**
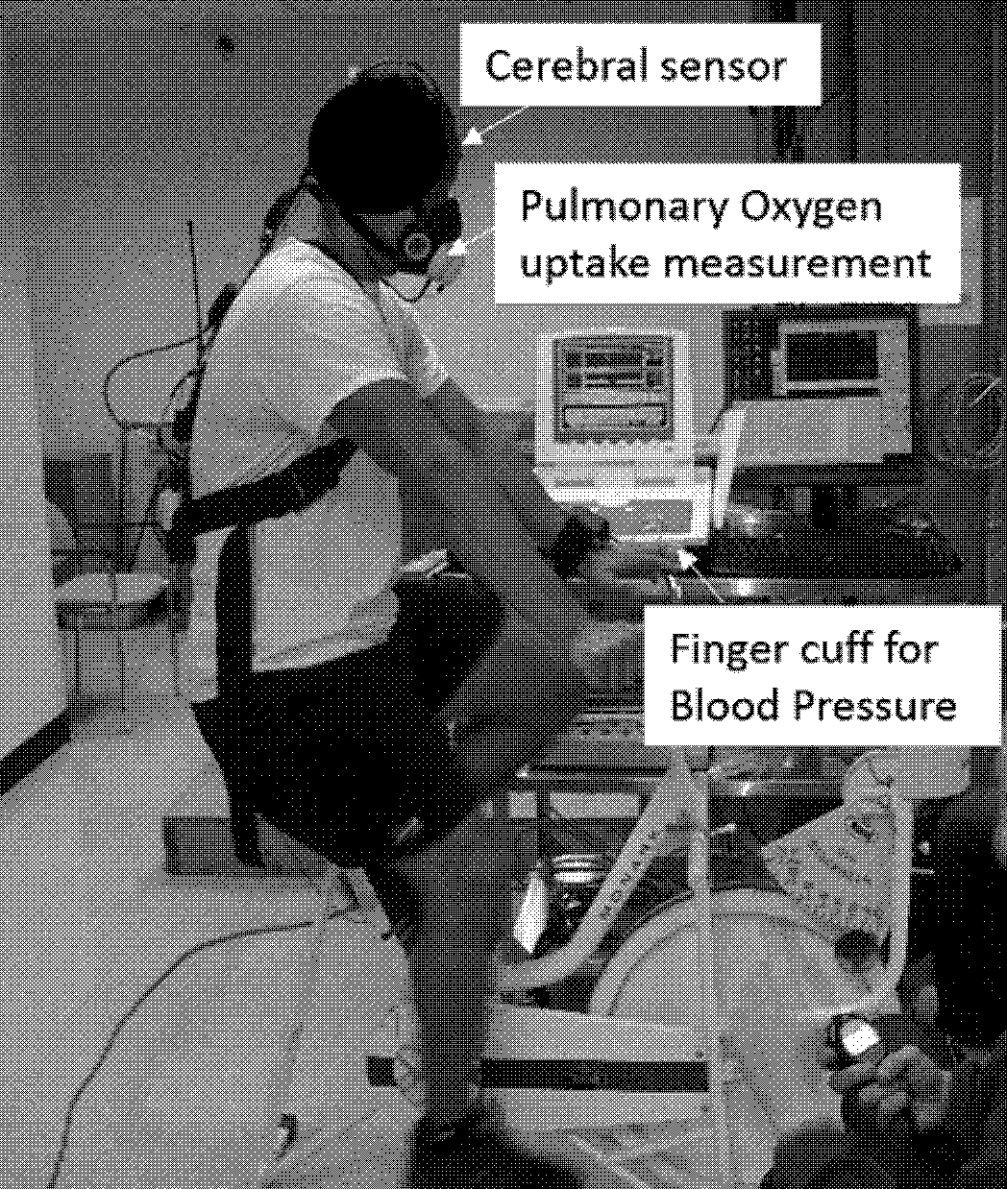
Experimental setup for the participant.

Participants completed a submaximal incremental cycling exercise on an ergometer (Monark Exercise, Sweden). Cycle ergometer exercise was selected as it does not require any external work on the body center of mass, and the resulting energy expenditure is dependent on a consistent level of mechanical work [2]. The seat height was adjusted so that there was a slight bend of the knee when the foot pedal was at its lowest point (Fig 1).

The participant began seated in a relaxed posture on the cycle ergometer for two minutes while baseline resting data were collected. The participant then pedaled at 60 revolutions per minute (rpm), beginning with no workload. The workload was increased by 0.5 kp every 2 minutes while the participant continued to pedal at 60 rpm. Prior to each workload increase, the participant was asked to provide a whole-body rating of perceived exertion based on the Borg scale ranging from 6–20 [15]. The task continued until the participant reached 60% of their age-predicted maximal heart rate. After task termination, the participant pedaled slowly at no load for a two-minute ‘active’ recovery period. The participant then remained seated on the cycle without pedaling for an additional two minutes of ‘passive’ recovery.

### Physiological Measurements

A portable wireless metabolic analyzer (K4b2, Cosmed, Italy) was used to measure breath-by-breath cardiorespiratory responses. The oxygen and carbon dioxide analyzers of the system were calibrated before each test with commercially available precision gases (16% oxygen, 4% carbon dioxide, and balance nitrogen). The flow meter was calibrated for volume using a 3 L syringe as per the manufacturer recommendations. Heart rate was recorded using a chest strap (Polar Electro Inc., Lake Success, NY) interfaced with the portable metabolic unit. Blood pressure was monitored using a cuff wrapped around the middle finger of the left hand (Finometer Pro, Finapres, Netherlands). Finometer also provides cardiac hemodynamic beat-to-beat parameters such as cardiac output (in L.min^-1^), stroke volume (in mL), and left ventricle ejection fraction (in mSec). Participants were instructed to keep their hands on the cycle handle throughout the submaximal work.

### Cerebral Hemodynamic Measurements

Participants were instrumented with two NIRS sensors (NIRO 300, Hamamatsu Photonics, Japan) on their forehead to continuously measure cerebral hemodynamic responses. Each optical sensor set consisted of an emitter and detector spaced 4 cm apart, and was placed over the left and right prefrontal cortex region, approximately 3 cm from the midline of the forehead and just above the supraorbital ridge [7]. Dark elastic bandage was wrapped around the NIRS sensors for light shielding. Changes in oxygenated (HbO_2_) and reduced (HHb) states of hemoglobin (Hb) were sampled at 2 Hz. NIRO 300 system also provides a tissue oxygenation index (TOI), defined as the ratio of HbO_2_/(HbO_2_+HHb) and is expressed in a percentage.

### Dependent Measures and Data Analysis

Physiological measurements were averaged over 10-second windows. Exercise duration was recorded as the task termination time after the participant reached the heart rate cutoff point. The workload at this specific time was recorded as the peak workload. Heart rate recovery (HRR, beats•min^-1^) was calculated as the absolute difference in HR at task termination and following 60 seconds of active recovery. Rate pressure product (in mm Hg.bpm) during baseline, submaximal work and recovery was calculated as the product of HR and systolic blood pressure. Total peripheral resistance (in dyn.Sec.cm^−5^) was calculated as the ratio of mean arterial pressure and cardiac output.

For the physiologic measurements, time points were normalized to task duration, and analysis was conducted at 20, 40, 60, 80, and 100% of task duration. Separate repeated measures analyses of variance were used to assess the effects of obesity, time, and their interaction on each of the gas exchange and cardiac and cerebral hemodynamic responses. Model assumptions for sphericity were assessed and a Greenhouse-Geisser correction was used as needed. Simple effects tests were used for post hoc analysis of significant interaction effects.

Assessment of the increase in absolute oxygen uptake (VO_2_) relative to the increase in HR was done based on linear regression models. Exponential relationships, in the form of $Y=b_{0}e^{-b_{1}X}$ (where *Y* = VO_2_ and *X* = time), between VO_2_ recovery and time were analyzed separately for each group using non-linear regression. Between-group differences in derived model parameters (*b*_*0*_ and *b*_*1*_) were evaluated using student’s t-tests. Pairwise correlation analysis was used to compare blood pressure and cerebral oxygenation responses. The level of significance for all analyses was set at *p*<0.05. The Statistical Package for Social Sciences (version 22) was used for all statistical analyses (SPSS Inc., Chicago, IL).

## Results

**Fig 2:**
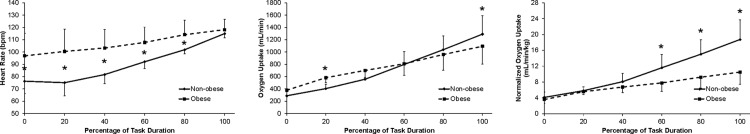
Physiological responses during incremental cycling scaled to the task duration. * represents a significant between-groups difference (*p* < 0.05) at the time point.

At task termination, the groups reached an average of 60.9 (3.3)% age-predicted maximum heart rate. However, the duration of the task in mean (SD) differed by group, with the non-obese terminating after 432 (67) seconds and the obese stopping after 330 (101) seconds (*p* = 0.016). All participants completed at least four minutes of cycling before reaching the stopping criteria. When gas exchange responses were normalized to task duration, the obese group exhibited higher VO_2_ compared to the non-obese group until 60% of task duration, at which point it dropped lower (see Fig 2B). The group level responses were significant at 20% and 100% of the task duration. VO_2_ per kg of body mass was equivalent between groups from 0% to 40% of task duration and then the non-obese group had more than 50% higher uptake (see Fig 2C).

The linear fit between HR and VO_2_ resulted in prediction equations of *VO*_2_ = −975.9 + 18.8*HR* for the non-obese group (R^2^ = 0.74) and *VO*_2_ = −161.38 + 8.55*HR* for the obese group (R^2^ = 0.20). The non-obese group had a higher rate of VO_2_ per beat and a more consistent linear relationship. Based on the adjusted prediction equations from Wasserman et al. [16], the non-obese group reached ~43% of their predicted VO_2max_ and the obese group reached ~34% of their predicted values (*p* = 0.036).

### Gas Exchange Responses

At baseline, absolute VO_2_ was similar between groups, however at termination of submaximal work, 13.5% reduced VO_2_ was observed in the obese group (*p* = 0.15, see Table 2). When VO_2_ was corrected for participants’ body mass, baseline values in the obese were 14% lower (*p* = 0.038), and at termination these values were 42% lower than the non-obese group (*p*<0.001). At baseline, VCO_2_ was similar between groups (*p* = 0.21) whereas it was higher in the non-obese group at termination (*p* = 0.06). Ventilation rate at baseline was similar between groups, and remained similar between groups at termination. Respiratory exchange ratio (RER) at baseline was similar between groups, however these values were higher in the non-obese group at termination (*p* = 0.01).

**Table 2:** Gas exchange responses in mean (standard deviation) at: baseline, termination of incremental submaximal cycling, and recovery.

Measure	Group	Baseline	At Termination	Active Recovery	Passive Recovery
Oxygen Uptake (mL•min^-1^)	Non-obese	239.6 (63.0)	1318.0 (302.1)	439.8 (172.6)*	257.4 (74.4)
	Obese	316.1 (52.3)	1138.7 (266.3)	630.4 (140.5)	303.9 (53.0)
Oxygen Uptake (mL•min^-1^•kg^-1^)	Non-obese	3.6 (0.8)*	18.5 (4.9)*	6.3 (2.1)	3.7 (1.2)
	Obese	3.1 (0.6)	10.8 (3.1)	6.0 (1.5)	2.9 (0.5)
Oxygen Uptake by BMI (ml•m^2^•kg^-1^•min^-1^)	Non-obese	11.2 (2.1)*	57.3 (13.5)*	20.0 (7.7)	11.4 (3.8)
	Obese	9.5 (1.7)	33.5 (9.6)	18.5 (4.2)	8.9 (1.7)
Oxygen Uptake by BAI (ml•min^-1^•%adiposity^-1^)	Non-obese	11.7 (2.1)	59.4 (13.3)*	21.2 (9.6)*	12.0 (4.3)
	Obese	10.6 (2.2)	37.1 (10.3)	20.4 (4.4)	9.8 (1.9)
Carbon Dioxide Output (mL•min^-1^)	Non-obese	233.0 (61.6)	1281.5 (322.1)	554.2 (171.3)	259.2 (76.4)
	Obese	282.1 (49.1)	1028.2 (250.8)	656.4 (134.0)	297.2 (51.7)
Ventilation Volume (L•min^-1^)	Non-obese	9.8 (1.4)	34.4 (11.3)	19.1 (6.2)	11.9 (3.2)
	Obese	11.2 (2.4)	30.8 (6.6)	23.1 (3.2)	12.9 (2.5)
Respiratory Exchange Ratio	Non-obese	0.91 (0.12)	0.99 (0.08)*	1.25 (0.20)*	1.09 (0.05)*
	Obese	0.87 (0.06)	0.90 (0.04)	1.04 (0.08)	0.99 (0.08)
Breathing Frequency (breaths•min^-1^)	Non-obese	15.5 (3.6)	21.8 (4.2)	19.0 (3.6)	18.7 (3.8)
	Obese	15.5 (3.9)	23.8 (6.3)	23.3 (4.9)	19.7 (3.7)

*Significant difference between non-obese and obese groups at *p* < 0.05

During active recovery, absolute VO_2_ was lower in the non-obese group (*p* = 0.029), whereas during passive recovery these values were similar between groups (*p*>0.05). During active and passive recovery, values of VO_2_ corrected for participants’ body mass was similar between groups (*p*>0.05). In addition, VCO_2_ and ventilation rate were similar between groups during both recovery periods. Respiratory exchange ratio was higher in the non-obese group during active (*p* = 0.004) and passive recovery (*p* = 0.005).

### Cardiac Hemodynamics

At baseline, HR was higher (by 24%) in the obese (*p* = 0.014), whereas these values were similar between groups at termination. Other responses such as cardiac output, stroke volume, blood pressure, oxygen pulse, rate pressure product, and left ventricle ejection fraction were similar between groups at baseline and task termination.

HR was higher in the obese group during both active (by 25%, *p* = 0.001) and passive recovery (by 25%, *p* = 0.01). Cardiac output was highest in the obese during both active (by 25%, *p* = 0.05) and passive recovery (by 26%, *p* = 0.03), however stroke volume was similar between groups. Rate pressure product was higher in the obese group during active (29%, *p* = 0.002) and passive recovery (25%, *p* = 0.01). Left ventricle ejection fraction was shorter in the obese during both active (12%, *p* = 0.03) and passive recovery (20%, *p* = 0.005).

During recovery, the rate of VO_2_ was slower for the obese group compared to the non-obese group when considering the parameters of exponential fit (Y_non-obese_ = 1258.7e^-0.0086(time)^ vs. Y_obese_ = 1091e^-0.0054(time)^_,_ *p*_b0_ = 0.24, *p*_b1_<0.001). HRR from the task termination to one-minute post task performance was double for the non-obese versus the obese (33.5(13.0) vs. 15.8(7.2), *p* = 0.0014). However, both groups returned to an equivalent level of heart rate compared to baseline after one minute (113.8(10.5)% vs. 112.0(15.1)%).

### Cerebral Hemodynamics

**Fig 3:**
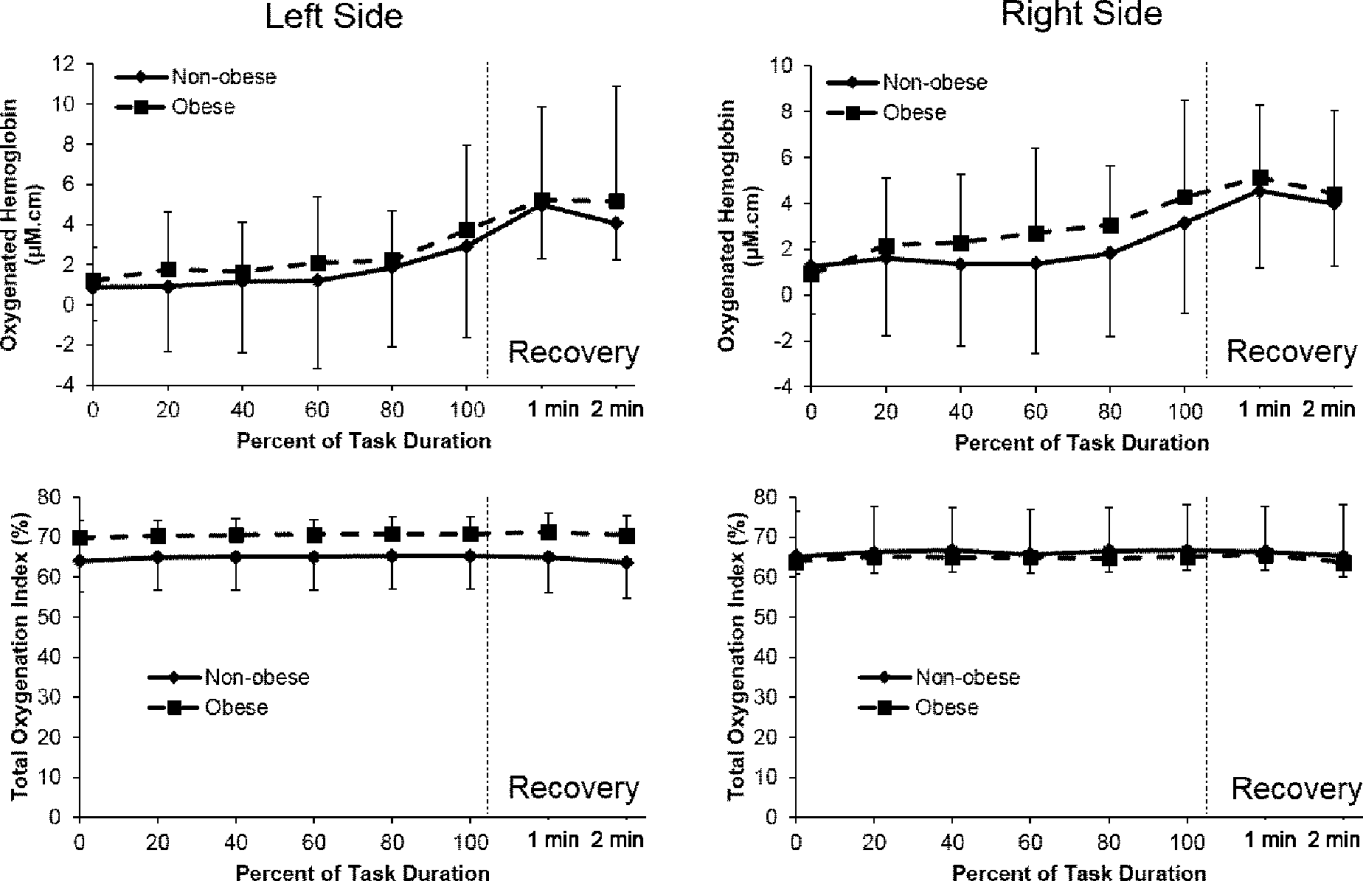
Prefrontal cortex oxygenated hemoglobin (HbO_2_; (a) left side, (b) right side) and total oxygenation index (TOI; (c) left side, (d) right side) in obese and non-obese groups during incremental submaximal cycling.

The trends in cerebral hemodynamics were consistent between groups and between both sides of the forehead (see Fig 3). Both groups exhibited an increase in oxygenated hemoglobin with increasing time normalized to task duration (*p*<0.001, Fig 3A and 3B). There was also a significant effect of time on the TOI (*p*<0.001, Fig 3C and 3D), however none of the measures showed significant group or group by time interaction effects. The correlations between systolic blood pressure and oxygenated hemoglobin were r = -0.38 (left region, *p* = 0.036) and -0.44 (right region, *p* = 0.016) for the non-obese group and r = -0.42 for both regions for the obese group (*p* = 0.031 and 0.033, respectively). Correlations between diastolic blood pressure and oxygenated hemoglobin were r = -0.72 (left region, *p*<0.001) and -0.49 (right region, *p* = 0.065) for the non-obese group while they were not significant for the obese group (r = -0.09 (left region, *p* = 0.67) and -0.17 (right region, *p* = 0.40).

## Discussion

This study examined the physiological responses between men of healthy weight and obese during a submaximal graded cycling task until participants attained their 60% of age-predicted maximum heart rate. The non-obese group cycled at a higher workload and for a longer duration than the obese group. The obese group had elevated baseline HR and reduced VO_2_ per body mass at baseline and task termination. Recovery of VO_2_ and HRR was slower in the obese group, while rate pressure product during recovery was higher in the obese group. However, both groups exhibited similar cerebral hemodynamics during recovery. These findings imply that irrespective of their low physiologic fitness, obesity does not impair myocardial performance and cerebrovascular function during sub-maximal exercise.

### Physiological Responses during Submaximal Exercise

Non-obese participants performed this moderate cycling work at a relatively higher workload (1.5 kp for the non-obese group vs. 0.94 kp for the obese) and for ~24% longer duration than the obese group. At task termination, factors such as an increase in body mass, BMI and BAI of the obese group resulted in lower VO_2_ than the non-obese group by 42%, 42%, and 38% respectively (Table 2), suggesting that although the same mechanical external work was performed by both groups, the increased demand of moving heavier legs during pedaling increases the cardiorespiratory demand for the obese participants [2]. The higher RER values at task termination in the non-obese group suggest the influence of longer duration of work as well as heavier workload. Ramos-Jimenez et al. [17] demonstrated higher VO_2_, but lower RERs at higher absolute workloads in their trained healthy participants compared to untrained participants. In the current study, the non-obese group were only recreationally active, thus considered as untrained participants. The RER indirectly represents fuel oxidation in terms of substrate utilization during exercise, where a ratio less than 1.0 indicates oxidation of fat whereas ≥1.0 indicates oxidation of carbohydrates. Therefore the lower RER at task termination in the obese might imply greater proportion of energy derived from fat oxidation in our heavier participants as compared to the non-obese. During moderate cycling exercise, Goodpaster et al. [18] demonstrated that obese sedentary men had increased rates of fat oxidation and reduced rates of carbohydrate oxidation compared to lean sedentary men.

The obese group had reduced fitness as evidenced by the elevated baseline HR and reduced VO_2_ per body mass at baseline and task termination, however oxygen pulse (a ratio of VO_2_ and HR) was similar between the groups. VO_2_ (a product of cardiac output and arteriovenous oxygen difference) and cardiac output (expressed as a product of stroke volume and HR) were also similar between groups at both baseline and task termination (Tables 2 and 3). Since oxygen pulse can also be expressed as a product of stroke volume and arteriovenous oxygen difference, such similarity in oxygen pulse might be due to compensatory change in arteriovenous oxygen difference between groups. Our findings were in contrast to Salvadori et al. [19], who observed a higher oxygen pulse in their obese individuals at low workloads, but not at high workloads of cycling until exhaustion.

**Table 3:** Cardiac hemodynamic responses in in mean (standard deviation) at: baseline, termination of incremental submaximal cycling, and recovery.

Measure	Group	Baseline	At Termination	Active Recovery	Passive Recovery
Cardiac Output (L•min-1)	Non-obese	6.2 (2.6)	10.6 (5.3)	7.4 (2.5)*	6.0 (1.9)*
	Obese	8.0 (2.0)	11.9 (3.5)	9.9 (2.7)	8.1 (1.7)
Stroke Volume (mL)	Non-obese	79.8 (30.4)	93.5 (41.0)	95.6 (26.6)	81.0 (23.6)
	Obese	83.4 (16.0)	103.5 (28.6)	95.4 (24.2)	86.1 (25.6)
Heart Rate (beats•min^-1^)	Non-obese	72 (12)*	115 (4)	76 (14)*	71 (10)*
	Obese	89 (20)	118 (9)	101 (16)	94 (20)
Oxygen Pulse (mL•beat^-1^)	Non-obese	3.4 (1.0)	11.4 (2.4)	5.8 (1.9)	3.7 (1.2)
	Obese	3.7 (0.9)	9.8 (2.6)	6.5 (2.0)	3.3 (0.9)
Systolic Blood Pressure (mm Hg)	Non-obese	134.0 (24.2)	149.0 (35.7)	127.0 (20.1)	130.6 (23.8)
	Obese	127.3 (15.4)	140.8 (27.2)	133.3 (20.7)	132.1 (15.2)
Diastolic Blood Pressure (mm Hg)	Non-obese	82.1 (15.9)	84.6 (20.7)	75.9 (15.9)	78.7 (14.3)
	Obese	80.1 (8.3)	86.4 (11.1)	82.0 (9.7)	83.3 (5.1)
Mean Blood Pressure (mm Hg)	Non-obese	104.6 (12.2)	110.9 (26.5)	97.4 (16.1)	98.9 (17.1)
	Obese	101.1 (11.1)	108.5 (16.1)	102.5 (13.5)	101.1 (7.1)
Rate Pressure Product (mm Hg•bpm)	Non-obese	9840 (2276)	17075 (4154)	9640 (2215)*	9316 (2109)*
	Obese	12053 (2908)	16701 (2760)	13629 (2704)	12374 (2255)
Total Peripheral Resistance (dyn•s•cm^−5^)	Non-obese	1639.3 (1399.2)	1219.1 (1588.4)	943.3 (564.2)	1167.3 (565.1)
	Obese	890.9 (357.5)	650.6 (236.3)	1071.6 (1275.9)	806.1 (229.5)
Left Ventricular Ejection Time (mSec)	Non-obese	277.2 (19.3)	266.0 (26.6)	296.1 (26.2)*	297.0 (25.0)*
	Obese	267.4 (53.8)	271.6 (17.2)	263.6 (32.7)	247.0 (38.4)

* Significant difference between non-obese and obese groups at *p* < 0.05

Obesity is associated with an increase in central blood volume and cardiac output to meet the metabolic demand of the adipose tissue, which is accompanied by an increase in stroke volume and a lowering of the peripheral vascular resistance [20, 21]. These cardiac responses, although increased from baseline during submaximal effort, were similar between groups due to the heart rate cut-off point compared to differences typically observed during a maximal effort until volitional exhaustion. It is reported that low ejection fraction is evident with obesity with depressed left ventricle fractional shortening, and is one of the factors for impaired myocardial contractility [22–24]. Although our obese group had ~4% lower left ventricle ejection time at baseline compared to the non-obese group, these values were not significant.

The influence of aerobic exercise on cardiovascular health in the obese is widely researched, however its role on cerebrovascular health in the obese is still in its infancy. One objective of the current study was to evaluate whether obesity-related differences would be seen in cerebral hemodynamics as a function of moderate workload. Both obese and non-obese participants in our study also evidenced an increase in cerebral oxygenation with increase in the moderate workload (Fig 3). During submaximal cycling, Ide et al. [25] demonstrated an increase in cerebral oxygenation with an increase in blood flow velocity in the middle cerebral artery, suggesting exercise-induced brain activation. In addition, cardiac output also influences cerebral blood flow during exercise [26]. An increase in cardiac output from baseline to task termination was observed (Table 3) suggests adequate cerebral perfusion as a function of moderate workload. These previous studies, along with the present findings, support cerebral vasodilation and thus an increase in cerebral blood flow in both groups during submaximal work, achieved partly through increases in NIRS-derived cerebral oxygenation mediated by an elevation in neuronal activity. Overall, the lack of between-group differences in cerebral responses during submaximal work indicate that the observed group-level differences in fitness were limited to anthropometric and gas exchange responses and did not occur at the cerebral level. As this task was not intended to fatigue the participants, the regulation of the prefrontal cortex, typically reported as being a pre-motor area responsible for movement planning [27], may not have been necessary in governing task performance.

### Recovery from Submaximal Exercise

The evidence of reduced fitness of the current obese group during task performance is further corroborated by the elevated HR and VO_2_ responses after two minutes of recovery. Recovery of VO_2_ was slower for the obese group, remaining over twice as high as the baseline values at the end of two minutes (634.6 mL/min vs. 306.7 mL/min). On the other hand, VO_2_ in the non-obese group returned to 40% above baseline, suggesting their aerobic power lead to faster recovery of phosphocreatine re-synthesis in the skeletal muscle as compared to compromised mitochondrial oxidative metabolism in the obese group [28]. This phenomenon of sustained elevated VO_2_ implies more oxygen is needed to completely oxidize fats than for carbohydrates. To this effect, the decrease in RER from active to passive recovery in both groups (Table 2) suggests a shift from carbohydrates to fat in substrate utilization, thereby preventing further depletion of muscle glycogen stores during recovery from exercise.

Rate pressure product was higher in the obese group during both active (by 29%), and passive recovery (by 25%). Rate pressure product is an indirect measure of the oxygen requirement of the myocardium and is a better predictor of myocardial metabolic demand than HR for both normotensive and patients with ischemic heart disease (e.g., Gobel et al. [29]). Previous studies have demonstrated that higher rate pressure product is influenced by obesity, and is considered as a determinant of cardiovascular risk since its increase precedes ischemic events [30]. Although other factors such as changes in ventricular volume and contractility state of the heart also influence myocardial VO_2_ (34), systolic blood pressure was similar between groups during recovery, suggesting a strong influence of HR on rate pressure product and thus myocardial metabolic demand in the obese group.

HRR following exercise has been identified as a risk factor for cardiovascular disease and all-cause mortality due to its relationship to vagal activity and reactivation [31, 32]. We used the value of HR recovery 1 min post task termination as an index of cardiac autonomic dysfunction, where rapid HRR is reported to be mediated by reactivation of cardiac vagal tone, thus influenced by the parasympathetic nervous system (e.g., Savin et al. [33]). Although HR was similar between our groups at the termination of exercise (Table 3), HRR was double for the non-obese versus the obese (an average of 34 vs. 16 bpm), implying faster recovery, thus higher fitness in the non-obese. Irrespective of the level of fitness, Gondoni et al. [34] also reported slower HRR in the obese as compared to that of participants with normal BMI. However, in the present study after one minute both groups returned to an equivalent level of heart rate compared to baseline (113.8% vs. 112.0%). Subsequent decrease in HR towards baseline after passive recovery is attributed to a combination of increasing vagal inhibitory effect and withdrawal of sympathetic drive, implying gradual weakening of the sympathetic system [32], as evidenced especially in our obese group. In the current study, the correlation coefficient between BMI and HRR was -0.73, which was consistent with that of Dimkpa and Oji [35] who reported the same coefficient of -0.73 between age-adjusted BMI and HRR. Similarly, Lins et al. [36] demonstrated impaired HRR after treadmill exercise with increased BMI, suggesting the obese group exhibits vagus nerve dysfunction. These authors defined ‘impairment’ as HRR ≤12 bpm, with these values three times more in the obese than in those with normal BMI.

Cardiac output was higher in the obese group compared to the non-obese during both active (25.3%) and passive (26%) recovery. Typically, stroke volume plays a major role in increase in cardiac output as compared to HR [37], however it was not significantly different between groups during recovery, implying the greater influence of HR on regulating cardiac output in both groups (Table 3). Since cardiac output can also be expressed as the ratio of VO_2_ over arteriovenous oxygen difference, higher cardiac output in the obese during recovery might also be due to elevated VO_2_ with transient increase in arteriovenous difference during the active recovery period. Such an elevated VO_2_ during recovery can also be due to the influence of increased lean and fat mass on the increased oxygen requirement by the obese, with large fat deposits in the obese generating a low-resistance vascular circuit, thus the increase in cardiac output to meet the needs of metabolic demand [38]. Impairment of left ventricle systolic function in the obese, in particular low ejection fraction is dependent on intrinsic myocardial contractile state, and is influenced by heart rate, left ventricle preload and afterload [39, 40]. In the present study, left ventricle ejection time was reduced in the obese during both active (by 12.3%) and passive recovery (by 20.2%), potentially suggesting left ventricular systolic dysfunction, thus influencing myocardial performance. This systolic interval measures the ejection of blood flow from the left ventricle to aorta, and is the time marked from the opening to closure of aortic valve. Shortening of ejection time is related to an increase in HR or an increase in left ventricular stroke volume [41, 42], and we observed a similar phenomenon in both groups, i.e., increase in HR with a simultaneous decrease in ejection time during both recovery periods, but stroke volume was similar between groups (Table 3).

Interestingly, NIRS-derived cerebral oxygenation and oxygenation index were similar between non-obese and obese groups during both recovery periods, suggesting that despite the obesity-related differences observed in cardiac hemodynamics, the cerebrovascular response was not influenced by the duration of recovery and intensity of submaximal exercise performance. Further, the equal duration of recovery for both groups was adequate for maintaining cerebral perfusion as evidenced by similar TOI between groups, implying fitness level does not influence brain health in the obese during and after submaximal exercise. Previous research suggests that an increase in exercise-induced cerebral blood flow depends on cortical activation, arterial blood pressure, cardiac output, and partial pressure of carbon dioxide [25]. However, cerebral blood flow is preserved by autoregulation, and remains constant between 60 and 150 mmHg of arterial pressure [43]. To this effect, although cardiac output was higher in the obese during both active and passive recovery, the mean arterial pressure in both groups was below 150 mmHg (Table 3), exhibiting autoregulation was intact in both groups during recovery.

### Potential Limitations

The sample was restricted to only male participants to minimize any potential within-group variability due to gender. This may limit the generalizability of the beyond males and comparison of the current results to previous studies that have considered only female participants. In addition, the cycling task was of reasonably short duration, particularly for the obese group, due to the cutoff of 60% age-predicted maximum heart rate. This resulted in low workloads, thus duration of the task and task demands may not have been of sufficient to observe group-level differences, especially in cerebrovascular responses. Although all participants indicated that they were recreationally physically active, the obese group may have been more deconditioned at the start of the experiment reducing their cycling time.

## Conclusions

While maximal aerobic capacity is often considered the key indicator of cardiovascular fitness, submaximal exercise tests are safer to perform and remain a valuable indicator of health risk and for evaluation of task demands. The obese group had reduced fitness as evidenced by the elevated baseline HR and reduced VO_2_ per body mass at baseline and task termination. Based on the RERs observed at task termination, we speculate a greater shift in carbohydrates as the prominent metabolic substrate over fat in the non-obese group. Cardiac hemodynamics and cerebral responses, although increased from baseline during submaximal effort, were similar between groups, suggesting that during submaximal work, irrespective of their low fitness, obesity does not impair myocardial performance and cerebrovascular function. Reduced fitness of the obese during task performance was further corroborated by the elevated HR and VO_2_ responses after the two-minute recovery. Both groups exhibited similar cerebral hemodynamics during recovery, implying restoration of cerebrovascular function. Understanding these group-level differences in fitness can allow for improved evaluation of abnormal responses to submaximal work, which is critical as individuals who are obese tend to be at higher risk of cardiovascular disease.
